# Editorial: Development of Microbial Ecological Theory: Stability, Plasticity, and Evolution of Microbial Ecosystems

**DOI:** 10.3389/fmicb.2016.02069

**Published:** 2016-12-21

**Authors:** Shin Haruta, Yasuhisa Saito, Hiroyuki Futamata

**Affiliations:** ^1^Department of Biological Sciences, Tokyo Metropolitan UniversityHachioji, Japan; ^2^Department of Mathematics, Shimane UniversityMatsue, Japan; ^3^Research Institute of Green Science and Technology, Shizuoka UniversityShizuoka, Japan; ^4^Department of Applied Chemistry and Biochemical Engineering, Shizuoka UniversityHamamatsu, Japan

**Keywords:** ecological theory, mathematical modeling, diversity, stability, evolution, interspecies interaction, resilience

We initiated this research topic based on the question “how can we develop microbial ecological theory?” We incorporated three important aspects, stability, plasticity, and evolution, which are key areas for progress in microbial ecological theory. Advances in microbial ecological techniques have allowed us to meticulously consider the dynamics of community structures, interspecies networks, and metabolic flux of microbial ecosystems. Studies using these techniques have demonstrated the stability (i.e., resistance, resilience, and persistence), dynamic equilibrium, and self-organizing ability of micro-ecosystems. Here, the fine contributors in this volume aimed to determine the central and general tenets of the ecological theory that describes the features of microbial ecosystems.

Analyses of the microflora in static-state conditions are insufficient to understand microbial ecosystems even if advanced omics techniques are used. Studies described in the included articles in this volume have tested several factors to elucidate how they affect the structures and functions of microbial communities. As external factors, the effects of temperatures (Melendrez et al.), nutrients (Aoyagi et al.; Aziz et al.; Itoh Vet al.; Kato et al.), and viruses (Kashiwagi et al.; Thingstad et al.) were intensively investigated. Internal factors, such as dormancy (Chihara et al.; Thingstad et al.), genetic modifications (Kashiwagi et al.; Melendrez et al.; Thingstad et al.), distribution (Chihara et al.; Melendrez et al.; Stegen et al.; Thingstad et al.), and interspecies interactions (Aoyagi et al.; Fatma et al.; Hanajima et al.; Hasegawa et al.; Kato et al.) were widely examined and discussed. Kashiwagi et al. and Melendrez et al. enlightened us about the genetic and physiological plasticity of microbes in microbial communities. The articles mentioned above successfully provided information that has not been observed in microbial communities, suggesting unknown characteristics of microbial ecosystems.

To unveil complex microbial ecosystems, it is desirable to strategically apply stimuli (i.e., perturbation) and comprehensively examine the microbial response. Mathematical modeling and simulations are powerful tools to develop a strategy to interpret comprehensive data and predict unknown characteristics of ecosystems (Chihara et al.; Fatma et al.; Haruta et al., 2013; Melendrez et al.; Stegen et al.; Thingstad et al.).

Relationships between the “*diversity*” and “*stability*” of ecosystems have been extensively discussed so far. Some researchers stated that high *diversity* is beneficial for high *stability*, whereas others stated that higher *diversity* reduces *stability*. Clear definition and evaluation scale for these claims are still lacking due to technical limitations. Omic analysis cannot completely grasp all elements of microbial communities, and the stability of the community must depend on the strength and duration of perturbations. Theoretical frameworks are important to generalize microbial ecological observations. Song et al. systematically redefined “resilience” as an intrinsic property of complex microbial ecosystems by integrating ecological and engineering concepts in their perspective article. Renslow et al. developed a new method that broadens the use of spatial data for assessing “resilience” in general and reported its applicability using a simulated microbial community. Ke and Miki comparatively summarized theoretical frameworks for close interactions between organisms and environments by reviewing studies on plant-soil feedback. Their review article provides various aspects for the development and applicability of ecological theories. In other words, these theoretical frameworks hopefully help to interpret big data from multi-omics approaches.

The development of sophisticated microbial ecological theory still has a long way to reach its goal. We believe that this volume is a constructive step toward expanding the frontiers of current microbial ecology. Furthermore, the articles in this volume propose the next step we will need for this long journey with many avenues for advancement. SH said the following; this volume reminds us that microorganisms are totally different from macro-organisms, i.e., microorganisms are physiologically versatile, show high heterogeneity even in clonal population, frequently change genetic elements, and have close cell-to-cell interactions. It may be necessary to recognize microbial ecosystems as a huge gene network, as proposed by Song et al. and refurbish ecological theories that have been established for macro-ecosystems. To discover a novel theory, I would like to put another question “are there any ecological theories applicable to complex microbial ecosystems?” YS said the following; it is crucial to study how parts of a system give rise to the collective behaviors of the system and how the system interacts with its environment to understand the features of microbial ecosystems. Developing mathematical something helpful (or as powerful as possible) for this problem has a quite long journey and is more challenging than developing those for macrobial ecosystems. HF said the following; the phrases of “population dynamics,” “community succession,” and “interspecies interactions” always make me imagine the breath of microbial ecosystems. Microbial ecosystems seemingly change with utter abandon; however, there should be any yet-to-be-discovered rules in their behavior. Through this volume, I recognize that consideration of time scales is indispensable to exactly understand the dynamics of microbial ecosystems. Mathematical modeling research of the resilience focusing on recovery time and length by Renslow et al. breaks through to approach to ecological theory. It has been developing to understand microbial ecosystems integrally by linking gene expression, metabolisms, and interspecies interactions with the aid of mathematics. I believe that the microbial ecological theory emerges through these integral analyses.

We acknowledge the talented group of researchers who have contributed to this volume. They have aggressively addressed the critical issues in microbial ecology and have presented research, which can be a foundation for future studies. While initiating this research topic, we aimed to gather diverse research articles and assemble all of them into a comprehensive picture representing the general rules of microbial ecosystems. Actually, however, it was difficult for us to sufficiently assemble the wide variety of the great achievements. We hope you enjoy this unfinished but vivid and entrancing picture and this volume will help to find other pieces of the puzzle obtaining complete picture of microbial ecosystems.

## Author contributions

SH drafted the manuscript. All authors wrote, revised, and approved the final version.

## Funding

This work was supported by KAKENHI Grant Numbers 15K12228, 26281038, and 26800080, Japan. It was also partially supported by ALCA project, Japan Science and Technology Agency.

### Conflict of interest statement

The authors declare that the research was conducted in the absence of any commercial or financial relationships that could be construed as a potential conflict of interest.
